# Modulation of Emotional Appraisal by False Physiological Feedback during fMRI

**DOI:** 10.1371/journal.pone.0000546

**Published:** 2007-06-20

**Authors:** Marcus A. Gray, Neil A. Harrison, Stefan Wiens, Hugo D. Critchley

**Affiliations:** 1 Clinical Imaging Sciences Centre, Brighton Sussex Medical School, University of Sussex, Brighton, United Kingdom; 2 Wellcome Trust Centre for Neuroimaging, Institute of Neurology, University College London, London, United Kingdom; 3 Institute of Cognitive Neuroscience, Alexandra House, University College London, London, United Kingdom; 4 Department of Psychology, Stockholm University, Stockholm, Sweden; University of Minnesota, United States of America

## Abstract

**Background:**

James and Lange proposed that emotions are the perception of physiological reactions. Two-level theories of emotion extend this model to suggest that cognitive interpretations of physiological changes shape self-reported emotions. Correspondingly false physiological feedback of evoked or tonic bodily responses can alter emotional attributions. Moreover, anxiety states are proposed to arise from detection of mismatch between actual and anticipated states of physiological arousal. However, the neural underpinnings of these phenomena previously have not been examined.

**Methodology/Principal Findings:**

We undertook a functional brain imaging (fMRI) experiment to investigate how both primary and second-order levels of physiological (viscerosensory) representation impact on the processing of external emotional cues. 12 participants were scanned while judging face stimuli during both exercise and non-exercise conditions in the context of true and false auditory feedback of tonic heart rate. We observed that the perceived emotional intensity/salience of neutral faces was enhanced by false feedback of increased heart rate. Regional changes in neural activity corresponding to this behavioural interaction were observed within included right anterior insula, bilateral mid insula, and amygdala. In addition, right anterior insula activity was enhanced during by asynchronous relative to synchronous cardiac feedback even with no change in perceived or actual heart rate suggesting this region serves as a comparator to detect physiological mismatches. Finally, BOLD activity within right anterior insula and amygdala predicted the corresponding changes in perceived intensity ratings at both a group and an individual level.

**Conclusions/Significance:**

Our findings identify the neural substrates supporting behavioural effects of false physiological feedback, and highlight mechanisms that underlie subjective anxiety states, including the importance of the right anterior insula in guiding second-order “cognitive” representations of bodily arousal state.

## Introduction

Recent theory distinguishes between two levels of emotional experience: phenomenology and awareness [1]–[2]. First-level phenomenology ‘core affect’ includes an integration of arousal (including physiological responses) with valence (pleasure-displeasure). At the second-level however, there is a conscious appraisal of these processes: Information from first-level physiological, expressive and experiential reactions is integrated with the behavioural context. Focusing on explaining phenomenology, earlier ‘peripheral’ models such as that of James and Lange argue that emotions arise from the interoception of physiological changes in the body [3]–[4]. A central contribution of physiological changes to emotion is also implicit in the two stage model of Schachter and Singer [5] in which it is argued that physiological arousal induced by adrenaline-injection can be interpreted as a state of either anger or elation depending on the concurrent context (an irritated or elated confederate) at a second-level of appraisal.

The importance of second-level appraisal mechanisms to emotional judgments is highlighted by false feedback experiments [6]. In the classic experiments of Valens [7], auditory feedback of participants' heart rate reactions was manipulated while male college students rated the attractiveness of photographs of naked women. When participants falsely believed their heart rate had changed (increased or decreased), they attributed greater attractiveness to the images. False feedback of either increased and decreased cardiac rate also influences emotional attributions [8], suggesting that any mismatch between ‘perceived’ and actual physiology confers greater salience to stimuli [9] perhaps by increasing attention to both viscerosensory information [10] and stimulus features [11]–[12]. While attentional enhancement may differentially influence emotional attribution to positive and negatively valenced stimuli [10], [13], emotional attribution to ambiguous stimuli may be particularly sensitive to false physiological feedback [14]–[16]. Such experiments illustrate a relationship between second level contextual interpretations and emotional attribution, yet there is relative paucity of research regarding the contribution of first level physiological arousal to these processes. Self-ratings of sexual arousal during erotic film viewing are reportedly increased after exercise, demonstrating that first level arousal may bias emotional attribution (termed excitation transfer) [17]–[18]. While the effects of false feedback are not mediated merely by physiological entrainment of arousal [10], [14]–[16], [19]–[21], the influence of actual physiological arousal on false feedback effects is poorly understood

Recent studies of interoceptive awareness [2], [22]–[25] strengthen the conception that higher-order representations of afferent viscerosensory information are tied to subjective emotional processing [26]. In primates, an evolutionarily-specialised spino-thalamo-cortical pathway is proposed to convey motivationally-salient bodily sensations centrally to insula cortex, by-passing obligatory mesencephalic relays present in rodents [27]. Studies of Pure Autonomic Failure (PAF) patients, in whom there is an inability to generate normal autonomic arousal responses as a consequence of sympathetic and parasympathetic effector denervation, suggest; 1) the presence of subtle emotional differences (i.e. are less emotional than control participants and less anxious than comparable neurological patients); 2) a general reduction in right insula activity, and; 3) attenuation of activity within amygdala and right insula cortex when processing threat stimuli [28]–[29]. In addition, neuroimaging evidence in healthy individuals suggests a specialized role of right anterior insula in conscious awareness of interoceptive state [23], [27], [30]. Thus, right anterior insula activity predicts individual differences in interoceptive sensitivity (performance of a heartbeat detection task [23]). In this task participants judged whether auditory tones were synchronous or asynchronous with their own heartbeat. In fact, during increased interoceptive demands, right anterior insula activity is greater during asynchronous feedback, suggesting that the region is tuned to mismatch between perceptual and visceral representations. The right anterior insula may support conscious awareness and appraisal of viscerosensory information in conjunction with orbital and dorsolateral prefrontal cortices [24], [26], [27], [30]. Anterior cingulate cortex may also contribute significantly, though neuroimaging experiences suggest that this region acts as a viscero-motor centre, for example in the initiation of sympathetic responses during behaviour [30]–[32]. Other regions also contribute to the interplay of bodily arousal states with cognitive and motivational processes, notably amygdala, somatosensory cortex and homoeostatic autonomic centres within the brainstem; i.e. periaqueductal grey matter (PAG), parabrachial nucleus (PN) and locus coeruleus (LC) [31]–[32].

In the present study, we examined neural activity associated with effects of false feedback of arousal state (heart rate) on emotional evaluations. Our approach differed from that of Valens [7] in that we did not give feedback as evoked reactions to stimuli rather feedback was presented in blocks representing ‘tonic state’. This approach permits temporal disambiguation of stimuli from feedback and reduces the potential for participants to alter ratings to meet implicit experimenter demands [33]. We also examined the processing of synchronous versus asynchronous cardiac feedback during periods of increased interoceptive demand. In addition we sought to examine the influence of first-level physiological arousal induced by isometric exercise [Bibr pone.0000546-Critchley3] [28 31] on both general emotional attributions, and on false feedback effects. We used functional magnetic resonance brain imaging (fMRI) to identify the neuroanatomical mechanisms through which these manipulations of both first and second-level physiological arousal can influence perception of emotional intensity of facial expressions. Stimuli were pictures of static facial expressions with positive (happy), negative (angry) and ambiguous (neutral) emotional valence. Our specific hypotheses were; first, that false feedback will alter ratings of face stimuli differentially across emotional facial expressions through engagement of regions including right anterior insula and amygdala. Second that during attributions with greater ambiguity (i.e. the emotional intensity of neutral relative to either angry or happy emotional expressions) right anterior insula activity will be more sensitive to asynchronous cardiac feedback, independent of heart rate.

## Results

### Behavioural results

**Figure 1:**
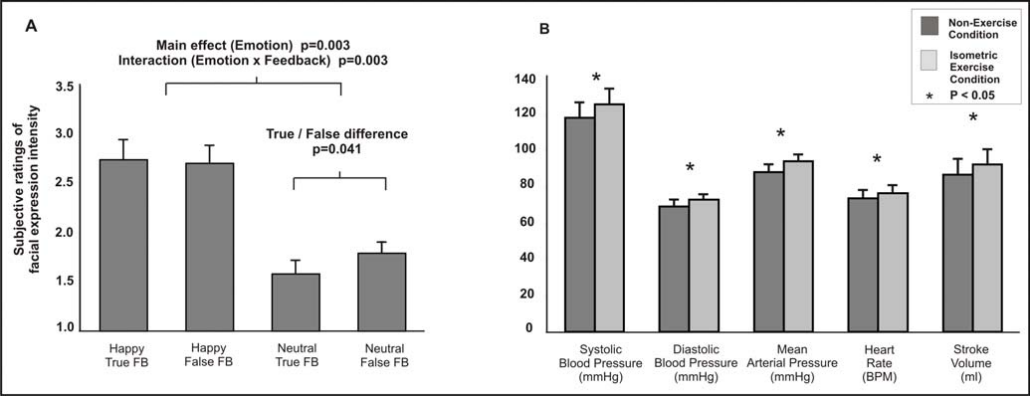
Subjective and physiological responses. (A) Mean subjective intensity ratings of happy and neutral facial expressions during both true and false physiological feedback of cardiac rate. Subjective intensity was rated on a four point rating scale via button box responses during functional scanning. A significant interaction between emotion (happy neutral) and feedback (true false), and a significant increase in the attributed emotional intensity of neutral faces during false relative to true feedback were observed. The volume of partial brain T2 data acquired is illustrated by the dashed box. (B) Peripheral physiological measures recorded outside the scanner showed that exercise increased systolic, diastolic, and mean arterial pressure, heart rate and stroke volume.

We first examined the influence of physiological feedback (true versus false) on behavioural ratings of intensity of face stimuli (portraying happy, angry, neutral facial expressions) in a repeated measures ANOVA. We observed a significant multivariate interaction of expression type and feedback on attributed emotional intensity [F(2,9) = 7.578, p = 0.012], in addition to a significant main effect of facial expression [F(2,9) = 9.90, p = 0.005]. Planned contrasts revealed a significant interaction between expression (happy neutral) and feedback (true false) [F(1,10) = 15.571, p = 0.003]. Further, the emotional intensity of neutral faces was rated significantly higher during false relative to true feedback [t(10) = 2.349, p = 0.041]. Across all conditions, the emotional intensity of neutral expressions was rated significantly lower than either angry [F(1,10) = 18.645, p = 0.002] or happy expressions [F(1,10) = 14.479, p = 0.003] see Figure 1.

We next examined the influence of isometric exercise in a repeated measures ANOVA, including the factors exercise (present absent) emotion (angry happy neutral) and feedback (true false). Exercise did not alter behavioural ratings of emotional expressions, and did not differentially influence false feedback effects.

### Peripheral physiological results

**Figure 2:**
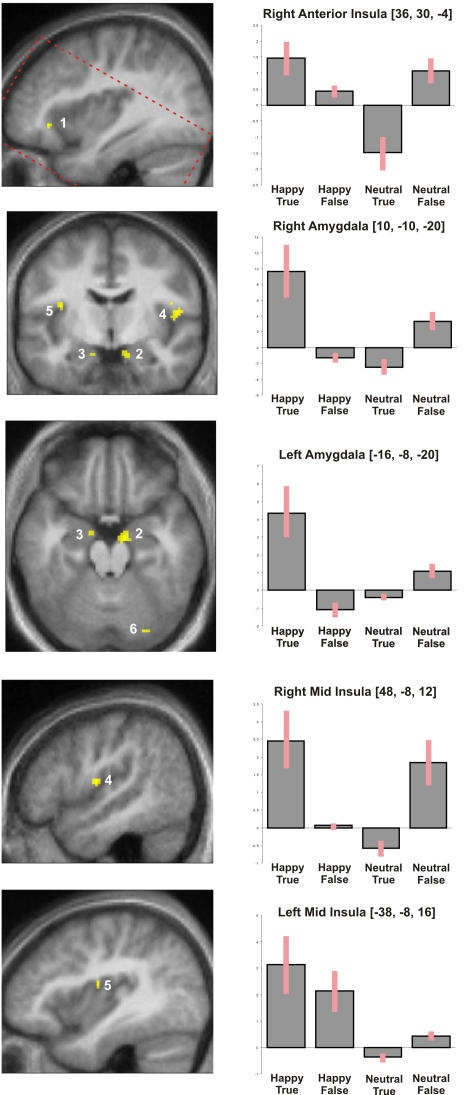
Neural Interaction of Emotion and Feedback Veracity. Left; Significant interactions of emotion (happy neutral) and feedback (true false) were observed on neural activity within; 1) right anterior insula, 2) right amygdala, 3)left amygdala, 4)right mid insula, 5) left mid insula and 6) posterior dorsal cerebellum. Functional interactions are projected onto a normalised mean structural image calculated from each individual subjects structural T1 weighted image. Dashed bounding box illustrates volume of acquired T2 data. Right; Contrast estimates at the illustrated regions are displayed.

Investigation of the beat-to-beat reconstructed brachial pressure waveform revealed significant effects of isometric exercise. In a repeated measures ANOVA of physiological measures across the blocked factors of feedback (true, asynchronous, false) and exercise (present , absent) isometric exercise was associated with significant increases in systolic [F(1,7) = 21.069, p = 0.003, partial η^2^ = 0.751] diastolic [F(1,7) = 19.122, p = 0.003, partial η^2^ = 0.732] and mean arterial pressure [F(1,7) = 29.696, p = 0.001, partial η^2^ = 0.809], stroke volume [F(1,7) = 5.584, p = 0.05, partial η^2^ = 0.444] cardiac output [F(1,7) = 17.620, p = 0.004, partial η^2^ = 0.716] and heart rate [F(1,7) = 9.843, p = 0.016, partial η^2^ = 0.584] relative to the no-exercise condition (Fig 2). Isometric exercise had no effect on measures of left ventricular ejection fraction, or total systemic peripheral resistance. Likewise, we observed no main effect of feedback and no interaction of feedback and exercise on measures derived from beat-to-beat reconstructed brachial pressure waveform.

### Functional neuroanatomical correlates

We next examined the neural activity corresponding to the observed interaction of feedback (true versus false) and expression (happy versus neutral). Specifically, we looked at activity which corresponded to greater increases in intensity ratings of neutral faces during false (relative to true feedback), compared to the same difference during ratings of happy faces; i.e. [(neutral false>neutral true)>(happy false>happy true)]. This interaction was observed in the right anterior insula cortex (Table 1 and Figure 2) and also in right mid insula cortex. Although other areas also showed a significant interaction, results suggested that left mid insula primarily responded more robustly to happy compared to neutral expressions (i.e., main effect). Moreover, the right and left amygdala responded to happy expressions more in the context of true (synchronous) feedback.

**Table 1:** Neural Interaction of Emotion and Feedback Veracity.

Region	Hemisphere	Co-ordinates	Number Voxels	t score
**Behavioural Interaction**				
Emotion (Happy/Neutral) vs Feedback ( True/False)				
Insula/frontal operculum	R	36, 30, −4	7	5.23
Mid insula (subcentral gyrus)	R	48, −8, 12	25	5.18
Mid insula (circular insula sulcus)	R	44, −8, 18	6	3.39
	L	−38, −8, 16	6	4.83
Amygdala	R	10, −10, −20	36	5.24
	L	−16, −8, −20	5	3.82
Superior temporal gyrus	L	−56, −20, 2	6	3.96
Lingual gyrus	L	−12, −70, −6	7	4.53
Inferior frontal sulcus	R	−24, 48, 2	7	4.26
Fusiform cortex	R	24, −80,−20	8	4.22
Medial thalamus	L	−6, −26, 6	5	4.11
Dorsal cerebellum	M	2, −72, −12	19	4.22

Neural activity corresponding to the observed behavioural interaction of feedback (true/false) and expression (happy/neutral) Activations are significant at p<0.001 (uncorrected) and activation extent greater than five contiguous voxels.

**Figure 3:**
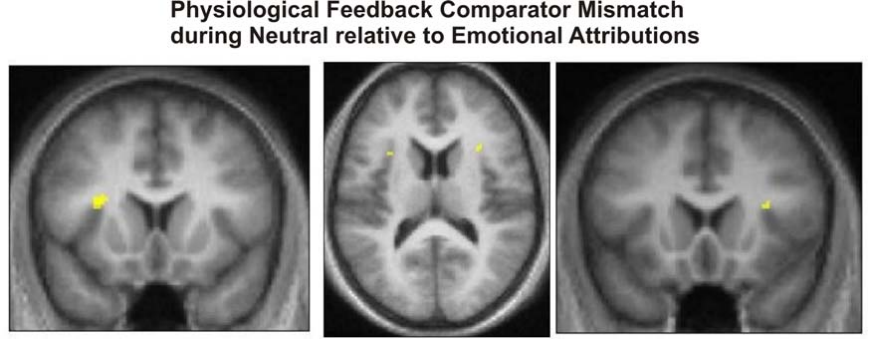
Neural activity associated with physiological comparator mismatch. During rating of neutral expressions, greater bilateral anterior insula activity was observed during asynchronous rather than synchronous cardiac feedback, relative to the same difference during ratings of happy expressions. Results are projected onto the normalised mean structural T1 weighted image.

We next examined evidence for a cardiac synchronous/asynchronous comparator mismatch function for the anterior insula cortex during interoceptive demand. Predicated on the on the notion that interoceptive information has greater salience when processing ambiguous (rather than intrinsically arousing) stimuli, we hypothesised that this would enable us to test for a comparator function of insula cortex in responding to mismatches in cognitive and interoceptive representations of physiological state, even at conditions of low bodily arousal. Thus, we specifically tested for a greater difference between asynchronous and synchronous cardiac feedback during the processing of neutral expressions, relative to the same difference during the processing of either happy or angry expressions; i.e. [ (neutral asynchronous>neutral true)>(emotional asynchronous>emotional true) ]. Significant interactions were observed in three regions; bilateral anterior insula cortex and dorsal cerebellum (Table 2, Figure 3).

**Table 2:** Neural activity associated with physiological comparator mismatch.

Region	Hemisphere	Co-ordinates	Number Voxels	t score
**Comparator Mismatch**				
Neutral (Asynch>True) & Emotion (Asynch<True)				
Insula (anterior circular sulcus)	R	30, 18, 14	5	3.89
	L	−32, 14, 16	26	5.00
Dorsal cerebellum	M	−2, −70, −6	7	4.16

Asynchronous physiological feedback (asynchronous>true feedback) evoked greater activity within insula and cerebellum during intensity rating of ‘ambiguous’ neutral expressions compared to high-intensity happy and angry expressions. Activations are significant at p<0.001 (uncorrected) and activation extent greater than five contiguous voxels.

As detailed above, exercise did not alter behavioural ratings of facial expressions-and showed no interaction with false feedback when rating neutral expressions. As a consequence we did not focus our investigations on exploration of neural correlates of non-significant behavioural interactions. Nevertheless, mapping of changes in first level representation within consciously accessible interceptive cortices, notably right anterior insula, was of direct interest.

**Figure 4:**
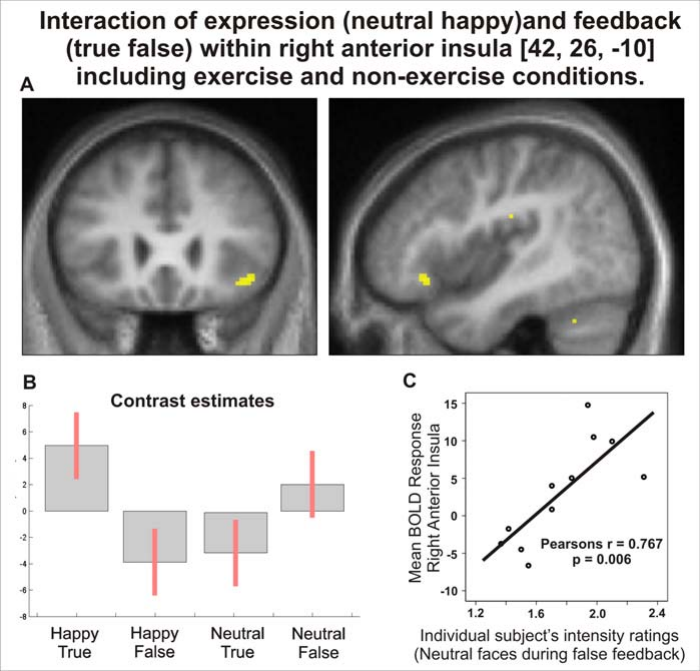
Interaction of Emotion and Feedback Veracity within Right Anterior Insula. Within the Right Anterior Insula, a significant interaction of emotion (happy neutral) and feedback (true false) was observed when considering both exercise and non-exercise conditions. This interaction closely resembles the right anterior insula interaction previously observed within the non-exercise condition alone. A) Coronal and sagittal sections, B) Contrast estimates. C) When considering both the exercise and non-exercise conditions, individual subjects' ratings of neutral faces during false is significantly correlated with BOLD responses within the right anterior insula cortex (across subjects).

In both exercise and rest conditions, the pattern of activity reflecting the influence of feedback veracity on rating of facial expressions was maintained, with significant interactions observed within right amygdala, bilateral posterior insula and right anterior insula. Within the right anterior insula cortex, the mean BOLD signal change in response to face stimuli portraying neutral expressions predicted individual differences in the magnitude of intensity ratings of these stimuli in the context of false feedback (Figure 4).

**Figure 5:**
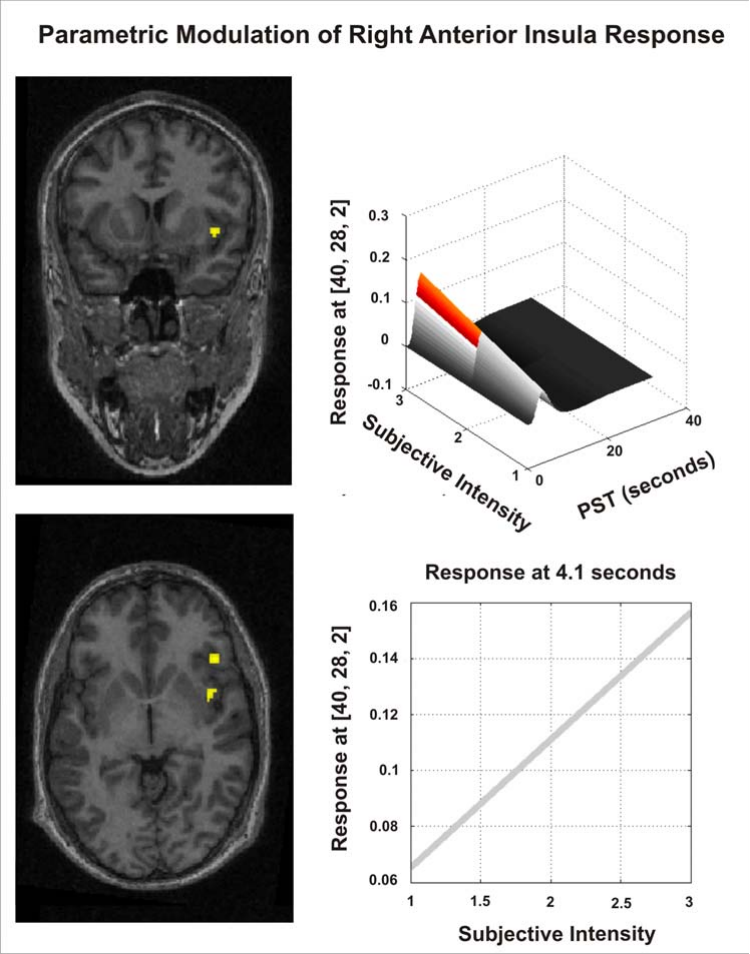
Right anterior insula and subjective ratings (within subject). Parametric modulation analysis of BOLD activity within right anterior insula by attributed intensity of neutral faces during false feedback were conducted within each subject. Second level group analysis of within subject results revealed a significant association within right anterior insula. Left: An individual subjects activation overlaid on their structural T1 weighted image. Right: Within the right anterior insula, the HRF amplitude is modulated as a function of the perceived intensity of neutral faces during false physiological feedback. The predicted response is illustrated across the entire HRF (right top) and at the peak HRF response (right bottom).

Pursuing the relationship between cerebral activity and false feedback induced biasing of emotional judgements, we used parametric analyses to examine within each participant the relationship between trial-by–trial behavioural ratings of neutral faces and regional BOLD activity. Again, within the right anterior insula cortex, we observed a significant association between BOLD activity and attributed intensity of neutral expressions during false feedback [T(1,36) = 4.10, p<001] (Figure 5). Increased activity within the right anterior insula cortex was associated with increased ratings of neutral expressions during false physiological feedback.

## Discussion

The present study demonstrates the influence of false feedback of physiological arousal on emotional appraisal of facial expressions through engagement of a discrete set of brain regions implicated in social and motivational behaviour. This neuroanatomical matrix encompasses brain regions that have been implicated functionally in the encoding of external emotional cues (STG, amygdala), representation of internal visceral information (insula cortex) and the contextual synthesis of emotional information for declarative awareness (anterior insula/operculum).

Our findings suggest a central integrative role for the anterior insula cortex. This region mapped differential influences of false feedback of arousal on differential processing of emotion, was sensitive to the timing of cardiac feedback (independent of arousal) and predicted within and across participants the degree to which behavioural ratings were altered during false physiological feedback. Together these novel data reveal neural substrates supporting the second level appraisal of emotional information, arising from an integration of perceptual processing within the context of physiological feedback representations, and influencing subjective affective judgement. Previous studies report right anterior insula engagement during conscious processing of emotional stimuli and in response to evoked physiological arousal responses [29]. Moreover, even in the absence of evoked arousal responses, right anterior insula activity can predict conscious awareness of interoceptive information and contribute to day-to-day experiences of anxiety symptoms [24]. Within a two-level theoretical model of emotion, the present data suggest right anterior insula as a likely neural substrate for second-level representation of subjective physiological arousal which in turn provides a reference for emotional processes including the attribution of intensity and salience to perceived social signals [2], [24], [27], [34]

False feedback of increased cardiac rate during rest enhanced the perceived intensity of neutral facial expressions but not angry or happy facial expressions. This is consistent with previous findings within the false feedback literature that the influence of physiological feedback is greatest during ambiguous judgements [14]–[16]. In our task, participants who rated neutral expressions as more intense exhibited increased activity within the right anterior insula. Moreover, at an individual level, intensity ratings of each stimulus were predicted by trial-by-trial changes in activity within this same region. One account for these observations is that right anterior insula supports a second-level consciously accessible emotional representation tailored toward contextual processing of emotional salience. The effect of false feedback on intensity ratings of neutral faces, mediated through right anterior insula, suggests that mismatch within hierarchical interoceptive representations enhances the salience of previously innocuous stimuli. Within the Paulus and Stein ‘insular view of anxiety’ [35] the interpretation is that bodily prediction error signals are attributed to ambiguous environmental stimuli and neutral faces become potential threats. Insula activation is observed during other conditions that engender emotional arousal, for example emotional recall [36]–[38] and appraisal of disgust stimuli [39]–[40]. The present study extends earlier findings [24], [29] to identify right anterior insula as a substrate through which cognitive processes are coloured by conscious representation of feelings. Interestingly, functional abnormalities with anterior insula cortex are observed in conditions such as alexithymia where there is impaired declarative awareness of subjective affect [41]. Anterior insula responses may reflect other character traits including neuroticism scores [42]. Future research could examine whether neuroticism infers greater susceptibility to false feedback effects within the right anterior insula.

Anatomically, Craig argues that the specialised contribution of right anterior insula in conscious interoception derives from a remapping of mid and posterior viscerosensory representations [27], [30]. Consistent with this logic, we observed that bilateral dorsal posterior insula activation reflects behavioural interaction at a phenomenological level. Within right mid insula, the veracity of physiological feedback clearly differentiated BOLD response to facial expressions, whereas within left mid insula BOLD response was more homogeneous within emotional categories. Mid and posterior insula regions contain an anatomically graded (viscerotopic) mapping of afferent signals, and provide an initial cortical representation of ongoing physiological activity, including autonomic reactions to emotive stimuli [26], [43]–[44]. Our observation that right insula differentiated between true and false feedback suggests that top-down influences on primary representations of cardiac physiology are more apparent within the right hemisphere [29].

We anticipated a contribution from amygdala to the emotional appraisal task. Patients with selective amygdala lesions show impairments in general social behaviour that correlate with deficits in emotional judgments of face stimuli [45]. Even in apparently healthy controls, the functional responsivity of the amygdala predicts individual differences in the accuracy of emotional judgment [46]. Thus, the amygdala may guide attentional processes underlying emotional attribution [47]. Most models ascribe to the amygdala a primary, phenomenological, level within the hierarchy of emotional processing, and amygdala responses can be evoked without the need for conscious appraisal [48]–[49]. This is consistent with a greater response to emotional (happy) expressions relative to neutral expressions. False physiological feedback promotes top-down re-evaluation of stimuli and interoceptive information, and was associated with a pronounced reduction of the primary amygdala responses observed during true feedback to emotive stimuli.

Behaviourally, false physiological feedback enhances attention to stimuli presumed to be physiologically arousing [6], enhancing the resources allocated to processing in sensory regions. The STG represents an extrastriatal region of visual cortex specialised for processing emotional expressions and other changeable aspects of face stimuli [50]–[51] in contrast to invariant properties such as identity, encoded within the fusiform face area (FFA) [52]. STG activity was modulated by false feedback in a manner that corresponded to the behavioural modulation of emotional intensity consistent with engagement of STG in enhanced top-down (attention-driven) processing of salience from faces [53].

Overall, we report the modulation of intensity judgements of face stimuli, particularly neutral faces by false feedback of physiological arousal state. Our neuroimaging data indicates engagement and interaction of three systems governing; first, initial representation and homoeostatic control of bodily arousal (insula); second, ‘automatic’ encoding of emotionally salient events (amygdala) and; third, visual and cognitive appraisal of stimuli (lateral temporal cortices). Further, our observations suggest a critical role for right anterior insula cortex in integrating information across these systems to support a second-level representation of emotionality that underpins subjective and behavioural experiences arising from false feedback. We did not observe any direct influence of first level physiological arousal on intensity judgements in the current study despite inducing significant increases in cardiovascular responses through isometric exercise. It is likely that misattribution of arousal is most probable where participants are unaware of the cause of their physiological arousal (i.e. excitation transfer effects). We did not observe misattributed arousal in the present study, presumably because first-level physiological arousal could be correctly attributed to isometric exercise.

The present study represents the first investigation of neural responses during both false feedback and exercise induced arousal. We observed an interaction of emotion and feedback on subjective measures, and a parallel interaction within regions implicated in cognitive appraisal, emotional processing and interoception. Our findings suggest an integrative contribution of right anterior insula cortex in second level representations of emotion that, in turn, predict individual differences in emotional behaviour. In sum, our findings provide insight into emotional appraisal mechanisms proposed in two-level theoretical models of emotion and identify the functional neuroanatomical substrate for hierarchical emotional appraisals of the social brain.

## Methods and Materials

### Participants

Twelve healthy right handed individuals (7 male, 5 female, age (mean 26.1 years±5.1 S.D.) gave informed written consent to take part in this study which was approved by the joint ethics committee of the Institute of Neurology and the National Hospital for Neurology and Neurosurgery, Queen Square, UK. Participants were screened to exclude psychiatric or systemic medical disorders and current medication usage.

### Task and procedure

Before scanning, each participant was familiarised with the task and procedures. Participants were informed that we were interested in the effects of different facial expressions on heart rate during exercise and rest. Individual heart beats were recorded via a pulse oximeter linked to the task computer and were audible as tones (pulses) through headphones. Participants were also informed that the tones (reflecting their individual heart beats) were essentially irrelevant, but were generated to allow us to measure heart responses. During questioning after the study, no participant said that this cover story raised suspicions.

**Figure 6:**
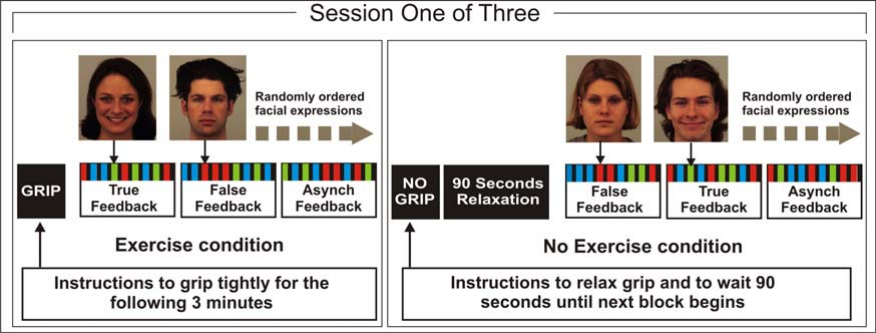
Experimental Paradigm and Task Design. Participants viewed emotional faces (happy, angry & neutral expressions, presented for 2 seconds each) and rated the emotional intensity during a subsequent blank screen (presented for 1 second). Within each of three sessions, participants performed the face rating task during isometric exercise and no exercise conditions (3 minutes each). Within each condition, physiological feedback of cardiac rate was true, asynchronous and false (1 minute each). Each participant completed three scanning sessions.

During the experimental task (Figure 6), a computer screen presented instructions (either to firmly clench or to relax hand grip), followed by emotional facial expressions from the Karolinska Directed Emotional Faces Set [54]. Each emotional stimulus category (happy, angry and neutral expressions) contained images of forty different identities (20 M, 20 F). Each image was displayed for two seconds. During the subsequent one second gap, participants rated the emotional intensity (four point scale) of the image via a left hand button press. In the right hand, the participant held a hand grip. Each participant completed three sessions and rated a total of 360 faces. In each session, the instruction screen first directed participants either to clench their hand tightly (exercise condition) or to relax their grip (no-exercise condition). Then, participants completed three minutes of the face rating task. For each of these three minutes, participants rated 20 faces while the nature of auditory feedback of heartbeats was manipulated (true, asynchronous, and false feedback). During *true* feedback, tones were presented synchronous with individual heartbeats. During *asynchronous* feedback, tones were delayed by half the inter-beat interval. During *false* feedback, no-exercise tones were presented at a rate ten percent faster than the preceding exercise heart rate, whereas exercise tones were presented at a rate 10 percent slower than the preceding no-exercise heart rate. Afterwards, participants completed another three minutes of the emotional appraisal task in the alternative exercise condition (either exercise or no-exercise) and with all three feedback conditions. The order of feedback and exercise conditions was randomized. In sessions where a no-exercise condition followed an exercise condition, a 90-second pause followed the instruction to relax grip, to allow exercise associated arousal to decline. At debriefing, participants were asked about their perceptions of heartbeat feedback. All participants were unaware (i.e. did not declare on direct questioning) of the manipulations of the auditory feedback of their heart rate (i.e., that it switched between synchronous *true, asynchronous*, and *false*). Further, participants confirmed that they remained focused on the face rating task.

### Data acquisition and image pre-processing

Functional echo-planar datasets sensitive to BOLD (Blood Oxygen Level Dependent) contrast were acquired at 1.5 Tesla (Siemens Sonata) The sequence, minimizing orbitofrontal signal dropout [55] achieved partial brain coverage (28 slices, 2 mm slice thickness, 1 mm gap, tilted −30° from inter-commissural plane, TR 2.52 seconds per volume, TE 40ms [see figure one]). After acquisition of the functional dataset, high resolution 1 mm^3^ T1-weighted structural scans were acquired from each participant.

Images were pre-processed using SPM5 (http://www.fil.ion.ucl.ac.uk/spm/), employing spatial realignment and sequential co-registration (6 parameter rigid body spatial transformation). Structural scans were segmented into CSF, grey and white matter images and iteratively normalized to standard space (Montreal Neurologic Institute) using a single generative model [56]. Transformation parameters for structural image segmentation and normalisation of were then applied to transforming participants' functional data into normalised images in standard space. Functional scans were subsequently smoothed with an 8 mm Gaussian smoothing kernel. The first 6 functional volumes were discarded to allow for equilibration of net magnetisation.

### Analytic design and data analysis

The analytic model embodied both blocked and event-related designs. ‘Exercise’ and ‘Feedback’ factors were blocked, to optimise development of physiological responses to exercise and psychological responses to the different types of cardiac feedback, respectively. Facial stimuli were modelled in a stochastic event-related manner to optimise design efficiency [57]. Within each feedback block, the pseudo-random presentation of facial stimuli was determined by event permutation [58]. Null events (15 percent of stimulus presentations) were also included to facilitate the identification of haemodynamic responses to stochastically ordered stimuli.

First level individualized design matrices were estimated for each participant. A canonical haemodynamic response function characterised the neural response to facial expressions during different levels of exercise and physiological feedback. Temporal derivatives were included in the first level design to reduce error variance associated with the timing of the haemodynamic response to stimuli and were not included in subsequent second level analysis. Movement regressors from the initial functional realignment were entered into the analytic model as covariates of no interest. Effects of feedback, facial expression and exercise on neural activity were computed on a voxel-wise basis for each participant in the form of Statistical Parametric maps (SPMs) of discrete contrasts within the general linear model. Subsequent second-level group random effects analyses were performed on the SPM contrast images of first level canonical HRF responses to permit formal inferences about population effects [59]. These group data are reported at a voxel-wise statistical threshold of P<0.001, with an additional constraint of cluster size (>5 contiguous voxels [60].

### Peripheral physiological responses

Peripheral physiological responses were also explored outside the scanner in an independent group of eight healthy right handed participants (5 male, 3 female, M age = 32.6 years, SD = 5.3 years) because recording equipment was not compatible with the fMRI environment. Participants were given identical task instructions and completed the experimental task while a finger cuff recorded beat-to-beat blood pressure with a Finometer (FMS, Finapress Medical Systems, and BV). This allowed a reconstruction of brachial artery pressure from cardiological measures were obtained, including arterial pressure at systole and diastole, stroke volume, cardiac output, left ventricular ejection time, total systemic peripheral resistance, and heart rate.
